# Pharmacological NF‐κB inhibition decreases cisplatin chemoresistance in muscle‐invasive bladder cancer and reduces cisplatin‐induced toxicities.

**DOI:** 10.1002/1878-0261.13504

**Published:** 2023-09-20

**Authors:** Rui M. Gil da Costa, Christine Levesque, Daniella Bianchi‐Frias, Payel Chatterjee, Hung‐Ming Lam, Carlos Santos, Ilsa M. Coleman, Pedro Ferreirinha, Manuel Vilanova, Nazaré Pinto da Cunha, Hugo Carvalho, Alexandra Moreira‐Pais, Ana Faustino‐Rocha, Tiago Neto, José Batista da Costa, Jonathan L. Wright, Rita Ferreira, Paula A. Oliveira, Joaquim Mendes, Margarida M. S. M. Bastos, Bruno Colaço, Carlos Lopes, Peter C. Black, Christopher J. Sweeney, Peter S. Nelson

**Affiliations:** ^1^ Division of Human Biology Fred Hutchinson Cancer Center Seattle WA USA; ^2^ LEPABE—Laboratory for Process Engineering, Environment, Biotechnology and Energy, Faculty of Engineering University of Porto Portugal; ^3^ ALiCE—Associate Laboratory in Chemical Engineering, Faculty of Engineering University of Porto Portugal; ^4^ Molecular Oncology and Viral Pathology Group, Research Center of IPO Porto (CI‐IPOP)/RISE@CI‐IPOP (Health Research Network) Portuguese Oncology Institute of Porto (IPO Porto)/Porto Comprehensive Cancer Center (Porto.CCC) Porto Portugal; ^5^ Center for the Research and Technology of Agro‐Environmental and Biological Sciences (CITAB) University of Trás‐os‐Montes and Alto Douro (UTAD) Vila Real Portugal; ^6^ Department of Urology University of Washington Seattle WA USA; ^7^ ICBAS University of Porto Porto Portugal; ^8^ CEDIVET Porto Portugal; ^9^ QOPNA University of Aveiro Aveiro Portugal; ^10^ Vancouver Prostate Centre University of British Columbia Vancouver BC Canada; ^11^ Department of Animal Science UTAD Vila Real Portugal; ^12^ INEGI, FEUP Porto Portugal; ^13^ Dana‐Farber Cancer Institute Boston MA USA; ^14^ Harvard Medical School Boston MA USA

**Keywords:** chemoresistance, cisplatin, muscle wasting, nephrotoxicity, parthenolide

## Abstract

Most patients with muscle‐invasive bladder cancer (MIBC) are not cured with platinum chemotherapy. Up‐regulation of nuclear factor kappa light‐chain enhancer of activated B cells (NF‐κB) is a major mechanism underlying chemoresistance, suggesting that its pharmacological inhibition may increase platinum efficacy. NF‐κB signaling was investigated in two patient cohorts. The Cancer Genome Atlas (TCGA) was used to correlate NF‐κB signaling and patient survival. The efficacy of cisplatin plus the NF‐κB inhibitor dimethylaminoparthenolide (DMAPT) versus cisplatin or DMAPT alone was tested *in vitro*. Xenografted and immunocompetent MIBC mouse models were studied *in vivo*. Platinum‐naive claudin‐low MIBC showed constitutive NF‐κB signaling and this was associated with reduced disease‐specific survival in TCGA patients. Chemotherapy up‐regulated NF‐κB signaling and chemoresistance‐associated genes, including *SPHK1*, *PLAUR,* and *SERPINE1*. In mice, DMAPT significantly improved the efficacy of cisplatin in both models. The combination preserved body weight, renal function, and morphology, reduced muscle fatigue and IL‐6 serum levels, and did not aggravate immuno‐hematological toxicity compared with cisplatin alone. These data provide a rationale for combining NF‐κB inhibition with platinum‐based chemotherapy and conducting a clinical trial in MIBC patients.

## Graphical abstract

NF‐κB up‐regulation promotes chemoresistance of muscle‐invasive bladder cancer (MIBC). The authors showed up‐regulated NF‐κB signaling in therapy‐naive MIBC subsets and post‐chemotherapy MIBC. Combining the NF‐κB inhibitor dimethylaminoparthenolide (DMAPT) with cisplatin therapy increased antitumor efficacy, and prevented cachexia and nephrotoxicity compared with cisplatin alone, without aggravating immuno‐hematological parameters. These data support the combination of NF‐κB inhibition with platinum‐based chemotherapy for MIBC treatment.

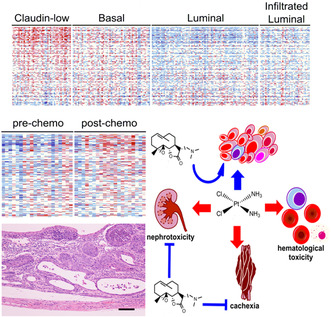

**Abbreviations**
ANOVA: analysis of variance
ATM: ataxia telangiectasia mutated
BBN: N‐butyl‐N‐(4‐hydroxybutyl)‐nitrosamine
BUN: blood urea nitrogen
CIS: *in situ* carcinoma
Ct: cycle threshold
DAB: diaminobenzidine
DMAPT: dimethylaminoparthenolide
ES: enrichment scores
FITC: fluorescein isothiocyanate
GEO: Gene Expression Omnibus repository
GSC: genomic subtyping classifier
GSVA: gene‐set variation analysis
H&E: hematoxylin and eosin
HBSS: Hank's balanced salt solution
IACUC: Institutional Animal Care and Use Committee
IHC: immunohistochemistry
IL‐6: interleukin‐6
MIBC: muscle‐invasive bladder cancer
MTT: 3‐(4,5‐dimethylthiazol‐2‐yl)‐2,5 diphenyl tetrazolium bromide
NEMO: NF‐κB essential modulator
NF‐κB: nuclear factor kappa light‐chain enhancer of activated B cells
OCT: optimum cutting temperature
PBS: phosphate‐buffered saline
PE: phycoerythrin
PerCP‐Cy5.5: peridinin‐chlorophyll protein‐cyanine5.5
qRT‐PCR: quantitative real‐time PCR
RBC: red blood cells
SAM: significance analysis of microarrays
TCGA: The Cancer Genome Atlas
TMA: tissue microarray
TURBT: transurethral resection of bladder tumor
UW: University of Washington
WBC: white blood cells
WNT16: Wnt family member 16

## 1 Introduction

Bladder cancer, most commonly presenting as urothelial carcinoma, may have a superficial distribution confined to the lamina propria and submucosa but in approximately 25% of cases, patients present with advanced‐stage disease with invasion of the muscularis mucosa or with metastatic disease [1]. Platinum‐based chemotherapy plays a prominent role in the care of patients with muscle‐invasive bladder cancer (MIBC): neoadjuvant chemotherapy followed by radical cystectomy or as part of chemoradiation, and is also a mainstay of therapy for patients with metastatic urothelial cancer. However, the clinical management of these patients remains a significant challenge, as only 15–25% show a complete response to therapy [2] and many patients are not candidates for cisplatin due to comorbidities such as renal insufficiency.

Urothelial cancer is a heterogeneous disease with different molecular gene expression subtypes. Five subtyping schemes have been proposed [3, 4, 5, 6, 7]. Importantly, molecular subtyping may help with more accurate prognosis and identify patients more likely to respond to chemotherapy [7]. For instance, the recently described claudin‐low subtype, which comprises approximately 10% of MIBC, is associated with poor outcomes and shows poor response to platinum‐based therapy [7, 8]. This subtype shows constitutive up‐regulation of the *nuclear factor kappa light‐chain enhancer of activated B cells* (NF‐κB) pathway, which has been implicated in chemoresistance and tumor progression in multiple cancer types including urothelial cancer [9, 10, 11]. Activation of NF‐κB in response to genotoxic stress is mediated by the ataxia telangiectasia‐mutated (ATM) protein through ubiquitination of the *NF‐κB essential modulator* (NEMO) [12]. NF‐κB drives chemoresistance through multiple mechanisms including the up‐regulation of anti‐apoptotic Bcl2‐family proteins [13] and induction of WNT ligands [14]. The development of NF‐κB inhibitors for treating patients with solid tumors remains an unmet need [15].

In this study, we assessed NF‐κB signaling in human bladder cancer samples before and after chemotherapy and assessed the efficacy and toxicity in preclinical bladder cancer models of an NF‐κB inhibitor dimethylaminoparthenolide (DMAPT) which has advanced to clinical trials. We observed that patients treated with platinum‐based chemotherapy showed up‐regulated NF‐κB signaling. In treatment‐naive patients, high NF‐κB activity was found in claudin‐low tumors and associated with poor disease‐specific survival. Based on these observations, we evaluated the efficacy of cisplatin alone versus a combination of cisplatin plus the clinical NF‐κB inhibitor DMAPT. We first confirmed the potential of this combination *in vitro* and then conducted *in vivo* tests, using a mouse xenograft model and an immunocompetent mouse model of MIBC induced by N‐butyl‐N‐(4‐hydroxybutyl)‐nitrosamine (BBN). In both *in vivo* models, the combination was significantly more effective than cisplatin alone. We also assessed how this combination could modify well‐known cisplatin toxicities. The addition of DMAPT reduced the expression of NF‐κB‐regulated genes in renal tissue and attenuated cisplatin‐induced nephrotoxicity. It also reduced circulating IL‐6 levels, preserved bodyweight, and reduced muscle fatigue. The combination treatment regimen did not induce significant differences in immuno‐hematological parameters.

## 2 Materials and methods

### 2.1 Patient samples

We evaluated tumor gene expression and clinical outcomes from patients with clinically T2–4 urothelial carcinomas who underwent platinum‐based neoadjuvant chemotherapy followed by radical cystectomy: 52 patients from the University of Washington and 223 from a multi‐institutional cohort, as previously described [7, 16, 17]. Pathologic response to chemotherapy was defined as complete response (pT0N0), partial response (pTa/pTIS/pT1N0), or non‐response (pT2‐4N0 and pT0‐4N1‐3) at cystectomy. Pre‐treatment (TURBT) and post‐chemotherapy radical cystectomy specimens from the 52 University of Washington patients were used to construct a tissue microarray (TMA). Replicate 1‐mm cores were taken from tumor, peritumor, and normal appearing surrounding stromal regions from both TURBT and cystectomy specimens. TURBT samples from 65 patients from the multi‐institutional cohort were used to build a second TMA. Matched pre‐ and post‐chemotherapy formalin‐fixed paraffin‐embedded tissue samples were used for whole transcriptome analysis using the GeneChip Human Exon 1.0 ST Array (Affymetrix, Santa Clara, CA, USA), and molecular tumor subtypes (luminal, luminal infiltrated, basal, and claudin low) were assigned according to a single‐sample genomic subtyping classifier (GSC) as previously reported [7, 16, 17]. Transcriptome data used in this study are available in the Gene Expression Omnibus repository (GEO) under accession number GSE87304. The study methodologies conformed with the standards set by the Declaration of Helsinki. The human ethics board of each institution approved this study and all patients provided written informed consent to analyze their tumor tissues. Protocol numbers: Bern, Switzerland KEK‐Be 219/2015; Vancouver, BC, Canada; H09‐01628; Southampton, UK, 10/H0405/99; Seattle, WA, USA, Fred Hutchinson Cancer Research Center, Washington, DC, USA: #7116; Amsterdam, The Netherlands, CFMPB‐104; UC Davis, Sacramento, CA, USA, 438935‐6; Rotterdam, The Netherlands, MEC‐2014‐642.

### 2.2 Transcript and gene set enrichment analysis

Transcript data were filtered to remove low expression genes (mean single‐channel normalized values less than zero). Differential expression between molecular subtype groups was assessed in r using the significance analysis of microarrays (SAM) samr package (R Foundation for Statistical Computing, Vienna, Austria). Genes were ranked according to *t*‐test statistics of group comparisons and used for GSEA on pathways within the Broad Institute's Molecular Signatures Database v. 6.1 [18, 19]. Radical cystectomy samples from complete and partial responders (pT0) were excluded from further analysis, resulting in 50 subtype‐classified TURBT, 20 of which had patient‐matched radical cystectomy samples from the UW cohort, and 223 subtype‐classified TURBT samples from the multi‐institutional cohort.

### 2.3 Survival analysis

RNA‐sequencing data from the TCGA muscle‐invasive bladder cancer cohort (6) were used to study the impact of NF‐κB signaling on patient survival. Processed RNA expression and clinical data for 408 samples were downloaded from the cBioPortal for Cancer Genomics [20, 21]. A total of 115 treatment‐naive patients who afterwards were treated with surgery and chemotherapy were further selected for additional analysis. Log_2_ RSEM values were used to compute sample‐specific pathway (enrichment) scores (ES) in r using gene‐set variation analysis (gsva) version 1.28.0 [22] for the pathways contained within the Broad Institute's Molecular Signatures Database v. 6.1. Pathway scores greater than 0 were categorized as “high” and those less than 0 as “low”. Patients were then stratified by these groups for Kaplan–Meier survival analyses and log‐rank tests for overall survival and disease‐free survival in r using survminer_0.4.3.

### 2.4 Leukocyte counts

Infiltrating leukocytes were manually counted using hematoxylin and eosin (H&E)‐stained preparations. Total leukocytes as well as neutrophils, macrophages, lymphocytes, and plasma cells were counted in 200× fields selected in areas representing the tumor–stroma interface at the tumor periphery. One or (when available) two fields were counted for each pre‐chemo and post‐chemo sample from each patient. Results were expressed as leukocytes per 200× field.

### 2.5 *In vitro* experiments

Two cell lines representing human urothelial carcinoma were used to test whether DMAPT enhances cisplatin's efficacy *in vitro*. UMUC3 (RRID: CVCL_1783) and HTB9 (RRID: CVCL_0126) cell lines were obtained from ATCC and cultivated in 96‐well plates *in vitro* using standard conditions at 5% CO_2_ and 10% fetal bovine serum in DMEM or RPMI, respectively. Cisplatin (1, 2.5, and 5 μm) and DMAPT (1, 2.5, and 5 μm) were dissolved in water and added to the cultures at serial concentrations. Drug combinations were tested at 1, 2.5, and 5 μm of both drugs. Cells were harvested at 48 and 72 h post‐exposure and counted manually. A 3‐(4,5‐dimethylthiazol‐2‐yl)‐2,5 diphenyl tetrazolium bromide (MTT) assay was used to assess cellular viability and metabolic activity. Both cell lines were authenticated using short tandem repeat profiling and tested negative for mycoplasma. The CompuSyn (ComboSyn Inc.) [23] was employed to determine whether the drug combinations showed additive or synergistic effects.

### 2.6 Xenograft model

The experiments were approved by the Dana‐Farber Cancer Institute Institutional Animal Care and Use Committee (IACUC) under protocol number 04‐111. Female 6‐week‐old nude mice (Crl:NU‐*Foxn1* ^*nu*^, Charles River) were injected subcutaneously in the left flank with 5 × 10^6^ UMUC3 cells per animal and tumors were allowed to grow over 4 weeks. By the 4th week, the animals were randomized into four treatment groups and started receiving cisplatin (group 1 *n* = 6, 5 mg·kg^−1^·week^−1^, i.p.), DMAPT (group 2 *n* = 6, 100 mg·kg^−1^·day^−1^, oral), cisplatin and DMAPT at the same doses (group 3 *n* = 6), or the saline vehicle (group 4 *n* = 5). The animals were housed in polystyrene cages under controlled temperature with 12 h/12 h light–dark cycles, were handled by trained researchers, and received daily health checks from veterinarians. Tumor volume was monitored using a caliper every 3 days. Tumor volume was calculated using the formula: volume = (length × 2.width)/2, where length is defined as the longest tumor diameter and width as the perpendicular diameter. Tumor ulceration or tumor volume > 2.0 cm were defined as humane endpoints. Results were expressed as the mean ± standard deviation of tumor volume per treatment group.

### 2.7 BBN bladder cancer model

Experiments were conducted at the University of Trás‐os‐Montes and Alto Douro (Vila Real, Portugal) animal facility, following approval by the local IACUC (the University of Trás‐os‐Montes and Alto Douro's Ethics Committee protocol approval number 10/2013) and in accordance with the European Directive 2010/63/EU on the use of laboratory animals. Female 6‐week‐old CD‐1 (Hsd:ICR) mice were obtained from Harlan (Barcelona, Spain). The animals were housed in polystyrene cages under controlled temperature with 12 h/12 h light–dark cycles. The mice were handled by trained researchers and received daily health checks from veterinarians. Animal weights were recorded individually every month and in groups every week. Food and water consumption were recorded weekly for each group. A mouse model of bladder cancer induced by *N*‐butyl‐*N*‐(4‐hydroxybutyl)nitrosamine (BBN, Sigma, St. Louis, MO, USA) was used to compare the efficacy of cisplatin versus cisplatin plus DMAPT against bladder cancer. One hundred female 6‐week‐old CD‐1 mice were randomly assigned to five groups (initial *n* = 20): group 1—untreated (negative control), 2—mice treated with BBN for 12 weeks at 0.05% v/v in drinking water (untreated cancer positive control), 3—mice treated with BBN for 12 weeks followed by a 6 weeks treatment with DMAPT (100 mg·kg^−1^·day^−1^, oral), 4—mice treated with BBN for 12 weeks followed by a 6 weeks treatment with cisplatin (Teva, Porto Salvo, Portugal) at 4 mg·kg^−1^ (intraperitoneal, i.p.) per week, 5—mice treated with BBN for 12 weeks followed by a 6 weeks treatment with DMAPT and cisplatin using the same dosing scheme of groups 3 and 4. Administration of cisplatin was always preceded by a hydration step where mice were administered saline (i.p., 1% of body weight). For groups 3 and 5, DMAPT administration started 24 h before the first cisplatin dosing. In the 13th experimental week, before starting the therapies, five untreated (group 1) mice and five BBN‐exposed mice (one from group 2, two from group 3, one from group 4, and one from group 5) were sacrificed, in order to confirm that the development of lesions was restricted to BBN‐treated animals. The final *n* for each group (15, 19, 18, 19, and 19 mice, respectively) was updated accordingly. One week after the final cisplatin dose, the animals were sacrificed for further analysis.

### 2.8 Histology and immunohistochemistry

Bladder lesions were separately classified by two pathologists (RGC and CL) blinded to the treatment groups. Disagreements were resolved by a joint slide review. Urothelial lesions were classified as urothelial hyperplasia, dysplasia (further divided into high‐ and low‐grade), squamous metaplasia, papilloma, *in situ* carcinoma (CIS), and invasive urothelial carcinoma (further classified as T1 or T2) (IARC, 2004).

For immunohistochemical studies, 5‐μm‐thick (or 8 μm for 53BP1) tissue sections were blocked with 3% hydrogen peroxide, underwent heat‐induced antigen retrieval in citrate buffer under pressure, and were probed with primary antibodies against 53BP1 (Abcam, Cambridge, UK ab36823 at 1 : 1000), Wnt family member 16 (Wnt16, Novus Biologicals, Centennial, CO, USA NBP1‐86403, 1 : 200), and interleukin‐6 (IL‐6, Santa Cruz Biotechnology Sc‐1265, 1 : 100) at room temperature. An avidin‐biotin detection kit (Vectastain Elite ABC kit, Vector Labs, Newark, CA, USA) was then employed according to the manufacturer's instructions. The slides were stained with diaminobenzidine (DAB) and counterstained with Mayer's hematoxylin. For 53BP1, at least 100 nuclei were scored, and the percentage of positive cells (arbitrarily defined as those containing at least three foci) was determined. For Wnt16 and IL‐6, an H‐score ranging between 0 and 300 was calculated by multiplying the percentage of positive cells by a factor that reflects staining intensity (0—negative, 1—weakly positive, 2—moderately positive, and 3—intensely positive).

### 2.9 *In vivo* renal toxicity of cisplatin and DMAPT

Mouse kidneys from the BBN study were weighed and fixed in formalin. The percentage of animals in each group with tubular damage (overt tubular necrosis or tubular degeneration with karyomegaly, clumped chromatin, and cellular tumefaction), glomerular damage (membranous, proliferative, or mixed), and interstitial nephritis (multifocal to coalescing mixed leukocytic infiltration) was determined histologically on H&E‐stained slides. Blood samples were used for measuring creatinine and blood urea nitrogen (BUN) (Rx Imola, Randox, Crumlin, UK). To further assess renal damage, and especially interstitial nephritis, we induced acute cisplatin damage [24, 25]. Male 6‐week‐old FVB/NJ mice, bred in‐house at the University of Trás‐os‐Montes and Alto Douro, were used for this purpose following IACUC approval (the University of Trás‐os‐Montes and Alto Douro's Ethics Committee approval number 10/2013). The mice were housed in polystyrene cages under controlled temperature with 12 h/12 h light–dark cycles, were handled by trained researchers, and received daily health checks from veterinarians. A single cisplatin dose (low: 4 mg·kg^−1^, high: 10 mg·kg^−1^, *n* = 5 per group) was administered i.p. Matched groups (*n* = 5) received cisplatin and 100 mg·kg^−1^·day^−1^ DMAPT orally, starting 24 h before cisplatin. The animals were sacrificed at 24 h and 2 weeks post‐administration. The kidneys were embedded in optimum cutting temperature (OCT) medium and stored at −80 °C. Blood samples were used to measure creatinine and BUN concentrations.

### 2.10 Quantitative real‐time PCR (qRT‐PCR)

mRNA was isolated from 8‐μm‐thick sections using the PicoPure RNA isolation kit (ThermoFisher, Waltham, MA, USA) and used to prepare cDNA. cDNA was generated from 1 μg of total RNA by using 2 μg of random hexamers for priming reverse transcription by SuperScript II (200 units per reaction; Invitrogen, Waltham, MA, USA). qRT‐PCR analyses were performed in triplicate using an Applied Biosystems 7700 sequence detector with approximately 5 ng of cDNA, 1 μm of designated primer pairs, and SYBR Green PCR master mix (Applied Biosystems, Foster City, CA, USA). The mean cycle threshold (*C*_t_) for each gene was normalized to levels of the mouse ribosomal protein S16 (*Rps16*) housekeeping gene in the same sample (Δ*C*_t_). Unpaired two‐sample *t*‐tests were used to determine differences in mean delta *C*_t_'s between treatment groups. *P* values <0.05 were considered significant. Primer sequences are summarized in Table S1.

### 2.11 Analysis of hematological and immunological parameters

While sacrificing experimental mice, blood was collected by cardiac puncture under anesthesia, into heparin‐coated tubes, and used for automated hematological analysis (Advia 120 Hematology System, Siemens, Munich, Germany). Red blood cells (RBC), hemoglobin, hematocrit, platelets, white blood cells (WBC), lymphocytes, neutrophils, and monocytes counts were expressed as mean ± standard deviation.

The thymus, spleen, and bone marrow cells from femurs were collected in Hank's balanced salt solution (HBSS), mechanically disrupted, and filtered through a 100 μm cell strainer to prepare single cell suspensions. Bone marrow cell suspensions were obtained by flushing the femoral axis with HBSS and similarly filtered. Collected cells were washed and resuspended in PBS supplemented with 1% BSA and 10 mm sodium azide. 1 × 10^6^ cells per sample were used for immunofluorescence staining using the following anti‐mouse monoclonal antibodies (all from BD Biosciences Pharmingen, San Diego, CA, USA): peridinin‐chlorophyll protein‐cyanine5.5 (PerCP‐Cy5.5)‐conjugated anti‐IgM (clone IL/41), phycoerythrin (PE)‐conjugated anti‐CD19 (clone ID3), PE‐conjugated anti‐CD3 (clone 145‐2C11), fluorescein isothiocyanate (FITC)‐conjugated anti‐Gr1 (clone RB6‐8C5) (for bone marrow cells analysis); FITC‐conjugated anti‐CD4 (clone RM4‐5) and PE‐conjugated anti‐CD8 (clone 53–6.7) (for thymocyte analysis); PerCP‐Cy5.5‐conjugated anti‐IgM (clone IL/41), PE‐conjugated anti‐CD19 (clone ID3), PE‐Cy7‐conjugated anti‐TCR‐β (clone H57‐597), FITC‐conjugated anti‐CD4 (clone RM4‐5), and PE‐conjugated anti‐CD8 (clone 53–6.7) (for spleen cells analysis). Flow cytometry data collection was performed in an EPICS XL flow cytometer using the expo32adc software (Beckman Coulter, Brea, CA, USA). Dead cells were excluded using propidium iodide (Sigma). Collected data were analyzed using flowjo v10.0.7 (Tree Star, Ashland, TN, USA).

### 2.12 Grip strength test

Just prior to sacrifice, mice from the BBN study were submitted to a grip strength test involving the forelimbs, as previously described [26]. The procedure was repeated six times for each animal and the highest force measurement was recorded as the maximum strength. Maximum grip strength values were divided by the animal's body weight and were presented as average ± standard deviation for each group. Muscle fatigue was calculated by comparing the last two contractions with the two first ones and was expressed as a percentage.

### 2.13 Statistical analyses

Continuous variables were analyzed using Student's *t* test. Differences among multiple groups were assessed using ANOVA with *post hoc* Tukey test, or Kruskal–Wallis test followed by Bonferroni correction as appropriate. Proportions were compared using Chi‐square tests followed by a *Z* test. Data were analyzed using prism 6.0 software (GraphPad Software, Boston, MA, USA). A *P* value < 0.05 was considered significant.

## 3 Results

### 3.1 NF‐κB signaling correlates with aggressive MIBC phenotypes

**Fig. 1:**
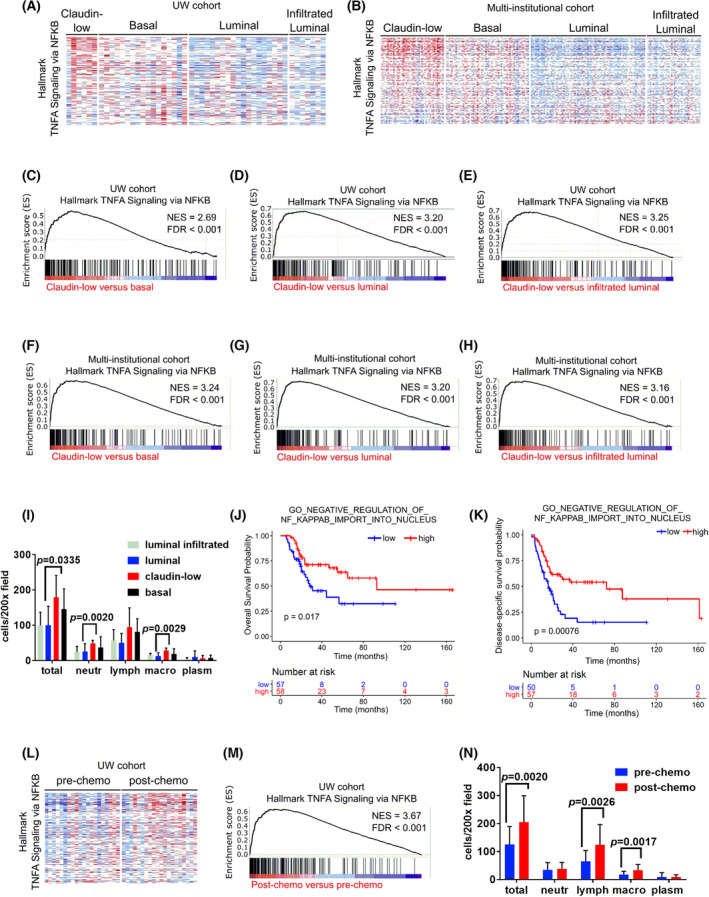
The NF‐κB pathway is up‐regulated in claudin‐low tumors and post‐chemotherapy muscle‐invasive bladder cancer (MIBC) samples. (A, B) Nuclear factor kappa light‐chain enhancer of activated B cells (NF‐κB) pathway genes expression in chemotherapy‐naive transurethral resection of bladder tumor (TURBT) samples representing different MIBC subtypes in independent patient cohorts: (A) patients from the University of Washington (UW), (B) patients from the multi‐institutional cohort, (C–H) gene‐set variation analysis (GSVA) enrichment plots comparing the Hallmark TNFα signaling via NF‐κB pathway between different MIBC subtypes in independent patient cohorts. FDR, false discovery rate; NES, normalized enrichment score. (I) Number of infiltrating leukocytes per 200× field in chemotherapy‐naive MIBC with different molecular subtypes, counted in hematoxylin and eosin (H&E)‐stained sections (average ± standard deviation). Differences were analyzed with unpaired two‐tailed Student's *t* tests. Lymph, lymphocytes; Macro, macrophages; Neutr, neutrophils; Plasm, plasma cells. (J–K) Overall (J) and disease‐specific (K) survival curves for 115 The Cancer Genome Atlas (TCGA) MIBC patients who received chemotherapy. Patients were stratified according to their NF‐κB GSVA enrichment scores (high = score >0; low = score<0) and differences were analyzed using a log rank test. (L, M) Hallmark TNFα signaling via NF‐κB pathway genes expression in matched pre‐ versus post‐chemotherapy MIBC samples from the UW cohort. (N) Number of infiltrating leukocytes per 200× field in chemotherapy‐naive and platinum‐treated MIBC, counted in H&E‐stained sections (average ± standard deviation). Differences were analyzed with unpaired two‐tailed Student's *t* tests. Lymph, lymphocytes; Macro, macrophages; Neutr, neutrophils; Plasm, plasma cells.

The claudin‐low subtype of MIBC is characterized by constitutive NF‐κB activation and responds poorly to platinum‐based therapy [7, 8]. We performed transcriptomic analyses followed by GSEA in two independent MIBC patient cohorts (the UW cohort and the multi‐institutional cohort) and determined that NF‐κB signaling was highly up‐regulated in claudin‐low compared to other molecular subtypes (Fig. 1A–H), confirming previous observations. The numbers of infiltrating leukocytes—especially neutrophils and macrophages—were significantly higher in claudin‐low tumors compared with other subtypes, consistent with up‐regulated NF‐κB signaling (Fig. 1I). We next used the TCGA cohort to confirm whether NF‐κB up‐regulation was associated with prognosis. When analyzing all the MIBC patients, no significant correlation between NF‐κB and outcomes was observed. However, when the analyses were restricted to the 115 patients who subsequently received chemotherapy (adjuvant or salvage) using multiple gene sets representing the NF‐κB pathway, we observed a highly significant association (*P* = 0.00076) with disease‐specific survival as well as a significant association (*P* = 0.017) with overall survival (Fig. 1J,K). These observations further support the hypothesis that NF‐κB plays a role in the response to chemotherapy and the prognosis of MIBC patients.

### 3.2 NF‐κB is up‐regulated in response to chemotherapy

We assessed whether acquired chemoresistance in other MIBC subtypes in response to platinum chemotherapy could be mediated by NF‐κB‐related mechanisms. We evaluated NF‐κB pathway activity in matching samples obtained before and after neoadjuvant platinum‐based chemotherapy from 20 patients in the UW cohort and observed that post‐chemotherapy samples classified pathologically as non‐responders showed significant NF‐κB up‐regulation by gene set enrichment analysis (GSEA) (Fig. 1L,M). Up‐regulated genes included several with known functions involved in cell survival and chemoresistance, such as *SPHK1*, *PLAUR*, and *SERPINE1*. Post‐chemotherapy samples also showed increased numbers of infiltrating leukocytes, specifically lymphocytes and macrophages (Fig. 1N), consistent with inflammatory responses driven by up‐regulated NF‐κB and in line with the transcriptomic data. These data suggest that chemoresistant (partial or non‐responder) tumors up‐regulate NF‐κB, regardless of their original molecular subtype.

### 3.3 DMAPT enhances the efficacy of cisplatin *in vitro* and *in vivo*

**Fig. 2:**
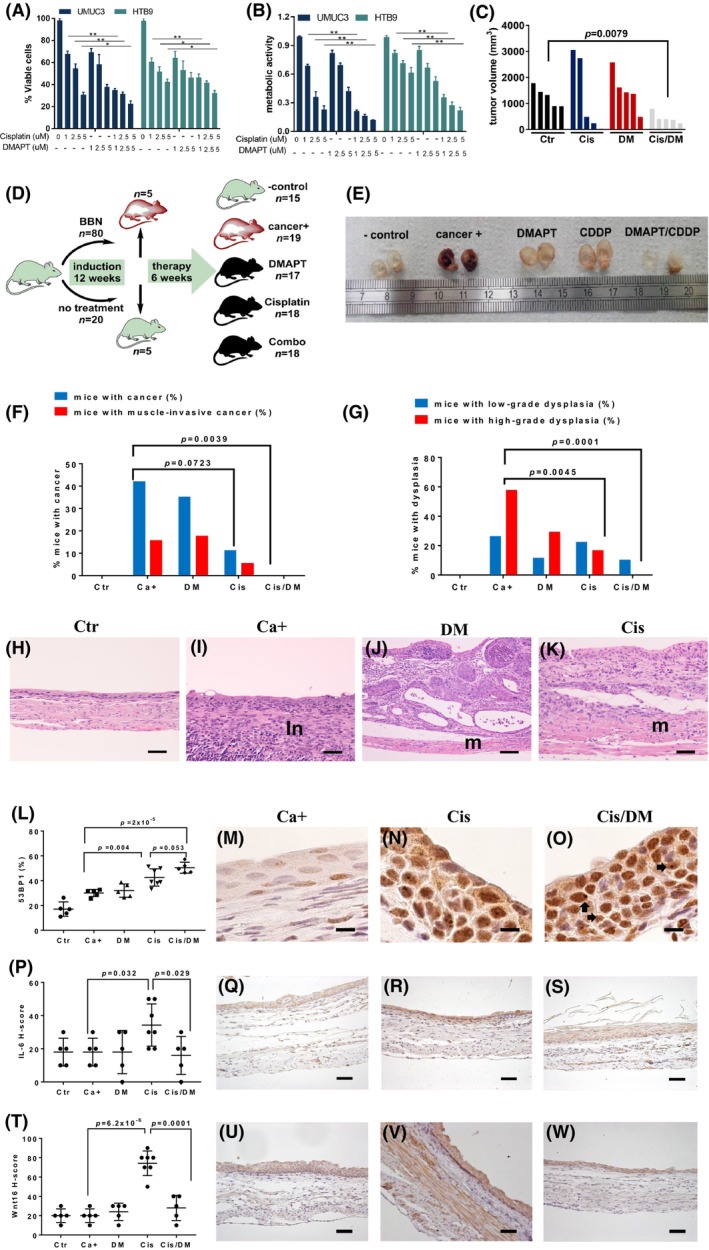
Dimethylaminoparthenolide (DMAPT) augments platinum activity against urothelial carcinoma. (A) *In vitro* HTB9 and UMUC3 cell viability (average ± standard deviation) using a Trypan blue exclusion assay in the presence of cisplatin, DMAPT, and their combinations, at 72 h (*n* = 3 replicas per experimental condition), *P < 0.05, **P < 0.001 using unpaired two‐tailed Student's *t* tests. (B) *In vitro* HTB9 and UMUC3 cell metabolic activity (average ± standard deviation) in the presence of cisplatin, DMAPT, and their combination, at 72 h, using a 3‐(4,5‐dimethylthiazol‐2‐yl)‐2,5 diphenyl tetrazolium bromide (MTT) assay (*n* = 3 replicas per experimental condition), **P < 0.001 using unpaired two‐tailed Student's *t* tests. (C) Tumor volume of xenografts formed by UMUC3 cells in nude mice 12 days after starting the treatment. Mice in each group received saline (Ctr, *n* = 5), 5.0 mg·kg^−1^ cisplatin i.p. weekly (Cis, *n* = 4), 100.0 mg·kg^−1^ oral daily (DM, *n* = 5), or their combination (Cis/DM, *n* = 5). Differences were analyzed using unpaired two‐tailed Student's *t* tests. (D) Schematic representation of the *N*‐butyl‐*N*‐(4‐hydroxybutyl)‐nitrosamine (BBN) experiment. Mice received BBN for 12 weeks, after which five BBN‐exposed and five control mice were sacrificed to confirm the development of pre‐malignant lesions. Surviving mice were divided into five groups: negative control (Ctr, *n* = 15), cancer‐positive untreated (Ca^+^, *n* = 19), DMAPT (DM, *n* = 17), cisplatin (Cis, *n* = 18), and combination (Cis/DM, *n* = 18), treated for 4 weeks and sacrificed. The *n* for each experimental group is valid for following panels (E–K); (E) Representative macroscopic view of the bladder following sacrifice, 24 h formalin fixation, and sagittal section (Ctr *n* = 15, Ca^+^ *n* = 19, DM *n* = 17, Cis, *n* = 18, and Cis/DM, *n* = 18). Ruler shows centimeters. (F) Percentage of mice with urothelial carcinoma (in gray) and with muscle‐invasive urothelial carcinoma (in black) for each experimental group, as determined in hematoxylin and eosin (H&E)‐stained sections (Ctr *n* = 15, Ca^+^ *n* = 19, DM *n* = 17, Cis, *n* = 18, and Cis/DM, *n* = 18). Differences were analyzed using Chi‐square tests. (G) Percentage of mice with low‐grade urothelial dysplasia (in gray) and with high‐grade urothelial dysplasia (in black) for each experimental group, as determined in H&E‐stained sections (Ctr *n* = 15, Ca^+^ *n* = 19, DM *n* = 17, Cis, *n* = 18, and Cis/DM, *n* = 18). Differences were analyzed using Chi‐square tests. (H–K) Representative H&E images (Ctr *n* = 15, Ca^+^ *n* = 19, DM *n* = 17, Cis, *n* = 18, and Cis/DM, *n* = 18) of: normal bladder from the Ctr group (H) (100×, bar = 100 μm), and high‐grade urothelial dysplasia from the Ca^+^ group (I) (200×, bar = 50 μm). Note intense inflammatory infiltrate (in) occupying the submucosa and muscularis tunics. Non‐muscle‐invasive urothelial carcinoma from the DM group (J) (100×, bar = 100 μm). Note intact muscularis tunic (M). (K) Muscle‐invasive urothelial carcinoma from the Cis group (200×, bar = 50 μm). Note invasion of the muscularis tunic (M); (L–O) Representative images of immunohistochemistry (IHC, *n* = 5 for Ctr, Ca^+^, DM, and Cis/DM, *n* = 7 for Cis) for the DNA damage marker 53BP1, diaminobenzidine (DAB)–Mayer's hematoxylin, 1000× bar = 10 μm. (L) Percentage of positive stromal cells for each experimental group (average ± standard deviation), differences were analyzed with unpaired two‐tailed Student's *t* tests; (M) Ca^+^ group, (N) Cis group, and (O) Cis/DM group. Arrows point to 53BP1 foci. (P–S) Representative images of IHC for interleukin‐6 (IL‐6) and DAB–Mayer's hematoxylin, 200× bar = 50 μm (*n* = 5 for Ctr, Ca^+^, DM, and Cis/DM, *n* = 7 for Cis). (P) H‐scores for IL‐6 IHC staining in each experimental group (average ± standard deviation), differences were analyzed with unpaired two‐tailed Student's *t* tests, (Q) Ca^+^ group, (R) Cis group. (S) Cis/DM group; (T–W) representative images of IHC for Wnt family member 16 (WNT16), DAB–Mayer's hematoxylin, 200×, bar = 50 μm (*n* = 5 for Ctr, Ca^+^, DM, and Cis/DM, *n* = 7 for Cis). (T) H‐scores for WNT16 IHC staining in each experimental group (average ± standard deviation), differences were analyzed with unpaired two‐tailed Student's *t* tests, (U) Ca^+^ group, (V) Cis group, and (W) Cis/DM group.

Following our observations that NF‐κB signaling is associated with chemoresistance in patient samples, we tested whether the NF‐κB inhibitor DMAPT could increase the anti‐cancer activity of cisplatin. For this purpose, we tested cisplatin and DMAPT as single agents and in combination against the human urothelial carcinoma cell lines HTB9 and UMUC3 *in vitro* and against UMUC3 xenografts in nude mice. We observed that while cisplatin and DMAPT as single agents showed moderate *in vitro* efficacy against these cell lines, their combination was significantly more effective at reducing cell viability and metabolic activity (Fig. 2A,B). The drug combination showed synergistic effects at the concentrations tested when analyzing metabolic activity data, but cell viability data indicate that additive effects are obtained at 2.5:2.5 μm and 5:5 μm doses (Table S2). *In vivo*, cisplatin and DMAPT as single agents did not consistently repress UMUC3 tumor growth compared with vehicle treatment (*P* > 0.05), while the combination of cisplatin and DMAPT significantly reduced tumor growth (*P* = 0.0079, Fig. 2C). However, no statistical differences were observed between each single agent and their combination (*P* > 0.05).

### 3.4 DMAPT enhances cisplatin's efficacy against MIBC in immunocompetent mice

We next evaluated the effects of cisplatin, DMAPT, and their combination in an immune‐competent *in vivo* model of urothelial carcinoma. We employed a mouse model of bladder cancer induced by the oral administration of *N*‐butyl‐*N*‐(4‐hydroxybutyl)nitrosamine (BBN), which closely recapitulates features of human MIBC of the basal molecular subtype [27]. To better mimic the genetic diversity of human populations, we employed outbred, immunocompetent CD‐1 mice, which were administered BBN for 12 weeks (Fig. 2D). Before treatment initiation, five BBN‐exposed and five control mice were sacrificed, and histological analysis showed that lesions were restricted to BBN‐exposed mice. Mice were divided into five groups, including a negative control, a BBN‐exposed but untreated group (cancer positive), and three groups that received DMAPT, cisplatin, or a combination of both over a six‐week treatment period. The bladder was then studied histologically, revealing that cisplatin alone was more effective than DMAPT at reducing the incidence of invasive cancers. However, cisplatin failed to significantly reduce the incidence of cancer compared with the untreated cancer‐positive group and allowed the development of lesions in two animals (11%), one of which showed muscle invasion. Importantly, the cisplatin plus DMAPT combination completely prevented bladder cancer in this model (*P =* 0.0039 compared with the untreated cancer‐positive group), as well as lesions of high‐grade dysplasia (Fig. 2E–K).

We next sought to confirm the involvement of the NF‐κB pathway in the response to therapy in this model. We confirmed that cisplatin induced DNA damage by a quantitative assessment of foci for the DNA damage marker 53BP1. Significantly more 53BP1 foci were identified in bladder tissues exposed to cisplatin compared to vehicle control (Fig. 2L). The cisplatin plus DMAPT combination caused more DNA damage than cisplatin alone, although the difference between the two groups did not reach statistical significance (*P* = 0.053; Fig. 2L–O). To confirm whether DNA damage correlated with up‐regulated NF‐κB signaling, we studied the expression of its downstream target gene *IL‐6*. Immunohistochemical scores for IL‐6 were significantly increased in cisplatin‐treated animals (*P* = 0.032 vs. the cancer‐positive group) and decreased in mice treated with the combination therapy (*P* = 0.029 cisplatin vs. cisplatin plus DMAPT) (Fig. 2P–S). Immunohistochemical scores for the NF‐κB downstream target gene *WNT16* followed a similar pattern: cisplatin increased WNT16 (*P* = 6.2 × 10^−6^ vs. the cancer‐positive group) and the combination therapy reduced it (*P* = 0.0001 cisplatin vs. cisplatin plus DMAPT) (Fig. 2T–W).

### 3.5 DMAPT prevents cisplatin‐induced renal damage

**Fig. 3:**
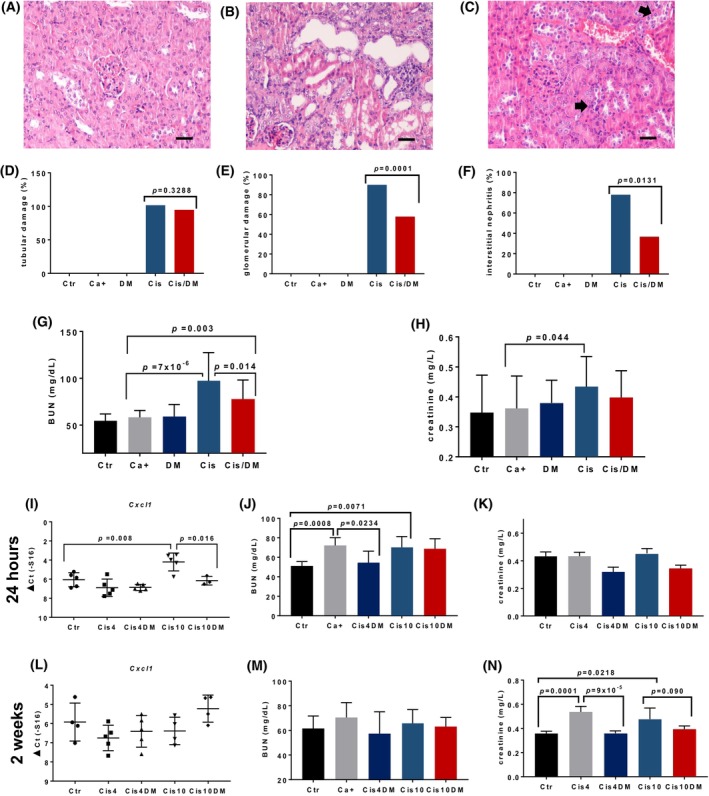
Dimethylaminoparthenolide (DMAPT) ameliorates cisplatin's nephrotoxicity in mice. (A–C) Representative hematoxylin and eosin (H&E) images of renal tissues (400×, bars = 20 μm) from: (A) Ctr group (*n* = 15) showing normal kidney histology; (B) Cis group (*n* = 18) showing tubular degeneration and interstitial nephritis; and (C) Cis/DM group (*n* = 18) showing preserved renal histology, with multifocal karyomegaly (arrows). Panels (D–H) show further data from all groups in the *N*‐butyl‐*N*‐(4‐hydroxybutyl)‐nitrosamine (BBN) experiments: (Ctr, *n* = 15), cancer‐positive untreated (Ca^+^, *n* = 19), DMAPT (DM, *n* = 17), cisplatin (Cis, *n* = 18), and combination (Cis/DM, *n* = 18). (D–F) Percentages of animals in each group (Ctr *n* = 15, Ca^+^ *n* = 19, DM *n* = 17, Cis, *n* = 18, Cis/DM, *n* = 18) showing (D) tubular degeneration/necrosis, (E) membranous or proliferative glomerulonephritis, and (F) interstitial nephritis. Differences were analyzed using Chi‐square tests. (G, H) Blood urea–nitrogen (BUN) and creatinine concentrations (average ± standard deviation) on experimental groups (Ctr *n* = 15, Ca^+^ *n* = 19, DM *n* = 17, Cis, *n* = 18, Cis/DM, *n* = 18). Differences were analyzed using analysis of variance (ANOVA). The acute toxicity of a single cisplatin dose (4 or 10 mg·kg^−1^) over renal tissues was then studied at 24 h (I–K) and 2 weeks (L–N) post‐administration. (I) Gene expression (average ± standard deviation) of *Cxcl1* for each experimental group at 24 h (Ctr—negative control, Cis4—4 mg·kg^−1^ cisplatin, Cis4DM—4 mg·kg^−1^ cisplatin and 100 mg·kg^−1^·day^−1^ DMAPT, Cis10–—10 mg·kg^−1^ cisplatin, and Cis10DM—10 mg·kg^−1^ cisplatin and 100 mg·kg^−1^·day^−1^ DMAPT, *n* = 5 per group); differences were analyzed with unpaired two‐tailed Student's *t* tests. (J, K) Blood urea and creatinine concentrations for each group (Ctr‐negative control, Cis4—4 mg·kg^−1^ cisplatin, Cis4DM—4 mg·kg^−1^ cisplatin and 100 mg·kg^−1^·day^−1^ DMAPT, Cis10—10 mg·kg^−1^ cisplatin, Cis10DM—10 mg·kg^−1^ cisplatin and 100 mg·kg^−1^·day^−1^ DMAPT, *n* = 5 per group, average ± standard deviation), respectively. Differences were analyzed using analysis of variance (ANOVA). (L) *Cxcl1* gene expression (average ± standard deviation) for each group at 2 weeks (Ctr—negative control *n* = 4, Cis4—4 mg·kg^−1^ cisplatin *n* = 5, Cis4DM—4 mg·kg^−1^ cisplatin and 100 mg·kg^−1^·day^−1^ DMAPT *n* = 5, Cis10—10 mg·kg^−1^ cisplatin *n* = 4, Cis10DM—10 mg·kg^−1^ cisplatin and 100 mg·kg^−1^·day^−1^ DMAPT *n* = 5); differences were analyzed with unpaired two‐tailed Student's *t* tests. (M, N) Blood urea and creatinine concentrations for each group (Ctr—negative control *n* = 4, Cis4—4 mg·kg^−1^ cisplatin *n* = 5, Cis4DM—4 mg·kg^−1^ cisplatin and 100 mg·kg^−1^·day^−1^ DMAPT *n* = 5, Cis10—10 mg·kg^−1^ cisplatin *n* = 4, Cis10DM—10 mg·kg^−1^ cisplatin and 100 mg·kg^−1^·day^−1^ DMAPT *n* = 5; average ± standard deviation), respectively. Ctr, Control; Ca^+^, mice with untreated cancer; DM, DMAPT; Cis, Cisplatin. Differences were analyzed using analysis of variance (ANOVA).

We next proceeded to study how the combination therapy could modify some well‐known toxicities induced by cisplatin. Nephrotoxicity is a major dose‐limiting cisplatin toxicity, and many cancer patients are not eligible for platinum therapy because of underlying renal dysfunction [28]. NF‐κB has been implicated in establishing a senescence‐associated secretory phenotype in response to chemotherapy which amplifies the initial renal damage [25, 28], and several NF‐κB inhibitors including parthenolide have been shown to reduce chemotherapy‐induced renal damage [29]. Histologically, in the BBN mouse model, we observed that mice treated with cisplatin alone showed severe renal pathology, with tubular degeneration and necrosis, membranoproliferative glomerular changes, and interstitial nephritis with multifocal to coalescing mixed leukocytic infiltration (Fig. 3A–F). The combination therapy partly prevented the occurrence of interstitial nephritis and glomerular changes but had no effect on direct tubular damage caused by cisplatin resorption. Histological changes were accompanied by functional consequences, as measured by blood urea (BUN) and creatinine concentrations (Fig. 3G,H). Mice treated with cisplatin alone showed significantly higher BUN concentrations compared with the cancer‐positive group (*P* = 7 × 10^−6^), and the combination therapy significantly reduced those values (*P* = 0.014 vs. the cisplatin‐treated group) without reaching baseline levels (*P* = 0.003 vs. the cancer‐positive group). Creatinine concentrations followed a similar pattern, although significant differences were found only between the cancer‐positive and the cisplatin‐treated group (*P* = 0.044). We next studied the expression of NF‐κB target genes in renal tissues, aiming to confirm the mechanism whereby DMAPT prevented the renal damage caused by cisplatin. However, inflammatory infiltrates in specimens from the BBN model chronically treated with cisplatin would likely show up‐regulated gene expression as a consequence of chronic inflammation rather than the early events that could be blocked by DMAPT. We therefore generated another mouse model to assess acute cisplatin effects. Mice were exposed once to cisplatin at a low (4 mg·kg^−1^) or a high (10 mg·kg^−1^) dose and sacrificed at 24 h or 2 weeks post‐exposure. Matched groups received DMAPT (100 mg·kg^−1^·day^−1^) while controls received saline. None of these animals showed histological changes, but blood urea and creatinine concentrations were elevated starting at 24 h and the cisplatin plus DMAPT combination countered these changes (Fig. 3I–N), further suggesting DMAPT could reduce cisplatin‐induced renal damage. The expression of 16 NF‐κB target genes including *Cxcl1*, *Cxcl5*, *Il1a*, *Il1b*, *Il6*, *IFNg*, and *Tnf* were measured by RT‐PCR (Fig. 3I,L, Table S3). Differences were observed at 24 h only, consistent with acute cisplatin‐induced damage (Fig. 3I–N). *Cxcl1* was specifically up‐regulated by cisplatin and the combination therapy with cisplatin and DMAPT blocked this response.

### 3.6 Immuno‐hematological toxicity of cisplatin and DMAPT

**Fig. 4:**
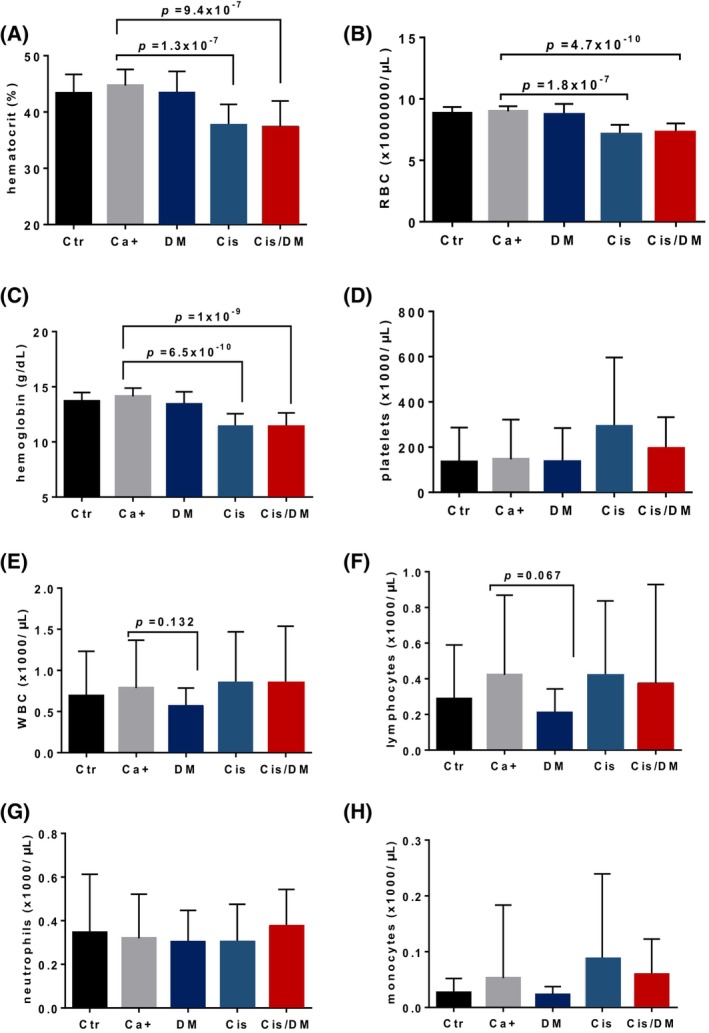
Dimethylaminoparthenolide (DMAPT) does not aggravate cisplatin's hematological toxicity in the *N*‐butyl‐*N*‐(4‐hydroxybutyl)‐nitrosamine (BBN) mouse model. Measurements of hematological parameters in BBN‐treated mice: negative control—Ctr *n* = 15, cancer‐positive untreated—Ca^+^ *n* = 19, DMAPT—DM *n* = 17, cisplatin—Cis *n* = 18, combination—Cis/DM *n* = 18. Hematological data are presented as average ± standard deviation in the following panels: (A) hematocrit values, (B) red blood cells, (C) hemoglobin, (D) platelets, (E) white blood cells, (F) lymphocytes, (G) neutrophils, and (H) monocytes. Differences were analyzed using analysis of variance (ANOVA).

Considering the well‐described toxicity of cisplatin toward hematopoietic progenitor cells and the key role played by NF‐κB in the biology of lymphocytes, we were concerned that an NF‐κB inhibitor might enhance cisplatin's immuno‐hematological toxicity to unacceptable levels. Mice treated with cisplatin alone showed a marked anemia, characterized by significantly reduced hematocrit, as well as lower red blood cell (RBC) counts and hemoglobin concentrations (*P* < 0.05, Student's *t* test), compared with the BBN and the negative control groups (Fig. 4A–C). The combination therapy did not significantly alter RBC counts compared with cisplatin as a monotherapy and no differences were observed concerning platelet counts (Fig. 4D). Cisplatin alone or in combination with DMAPT did not reduce white blood cell (WBC) counts (Fig. 4E). However, mice treated with DMAPT alone displayed a reduction in WBC, which was largely due to a near‐significant lymphopenia (*P* = 0.067, Student's *t* test; Fig. 4F). No significant differences were observed concerning neutrophils or monocytes (Fig. 4G,H). This prompted us to undertake a detailed study of both primary and secondary lymphoid organs—the bone marrow, thymus, and spleen—in all experimental groups (Fig. S1). Flow cytometric analysis of these organs showed that cisplatin alone or in combination with DMAPT was associated with reduced B lymphocytic (CD19^+^, IgM^+^, or IgM^−^) populations in the bone marrow, as well as with severe thymic and splenic atrophy. No adverse effects were observed against Gr1^+^ myeloid cells in the bone marrow. DMAPT alone did not induce significant differences in any of the granulocytic or lymphocytic cell populations studied in any of the organs.

### 3.7 DMAPT prevents cisplatin‐induced cachexia

**Fig. 5:**
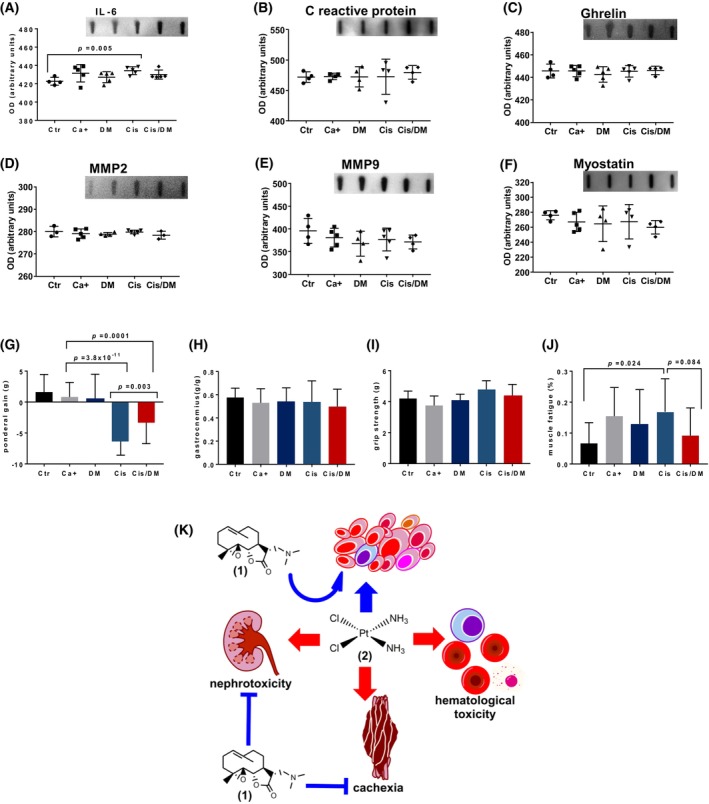
Dimethylaminoparthenolide (DMAPT) reduces weight loss in the *N*‐butyl‐*N*‐(4‐hydroxybutyl)‐nitrosamine (BBN) model of muscle‐invasive bladder cancer (MIBC). The concentration of markers related to muscle wasting was studied in blood serum using dot blots. (A–F) represent average ± standard deviation for interleukin‐6 (IL‐6), C reactive protein, ghrelin, matrix metalloproteinase 2 (MMP2), matrix metalloproteinase 9 (MMP9), and myostatin for each experimental group (negative control—Ctr *n* = 15, cancer‐positive untreated—Ca^+^ *n* = 19, DMAPT—DM *n* = 17, cisplatin—Cis *n* = 18, combination—Cis/DM *n* = 18), respectively. Differences were analyzed with unpaired two‐tailed Student's *t* tests. Next, we evaluated muscular morphological and functional parameters for each group (Ctr *n* = 15, Ca^+^ *n* = 19, DM *n* = 17, Cis, *n* = 18, Cis/DM, *n* = 18) (G–J). Differences were analyzed using analysis of variance (ANOVA). (G) Ponderal weight gain following therapy for each group (average ± standard deviation). (H) Gastrocnemius muscle weight over body weight (average ± standard deviation). A grip strength test was conducted before sacrificing the animals and six consecutive strength measurements were obtained for each animal. (I) Maximum grip strength (average ± standard deviation) after correcting for body weight. (J) Muscle fatigue, calculated as a percentage between the last two contractions and the two first ones. (K) Diagram representing how DMAPT (1) augments the efficacy of cisplatin (2) against MIBC while reducing its nephrotoxicity and pro‐cachectic effects.

Cisplatin is known to aggravate cancer cachexia in a significant proportion of patients, a phenomenon thought to be partially mediated by IL‐6 and other pro‐inflammatory cytokines [30]. Since DMAPT was able to reduce the expression of IL‐6 induced by cisplatin in the bladder stroma, we hypothesized that it could also reduce its circulating levels and counter its pro‐cachectic effects. We performed slot blot analyses of blood plasma samples from all experimental groups for inflammation markers—C reactive protein and Il‐6—as well as other factors thought to be involved in cancer cachexia—ghrelin, myostatin, and matrix metalloproteinases (MMP) 2 and 9 (Fig. 5A–F). No significant changes were observed for any of these markers, except for IL‐6. Mice treated with BBN showed a moderate increase in circulating IL‐6, which reached statistical significance (*P* < 0.05) in cisplatin‐treated mice. The levels of IL‐6 in mice treated with the combination therapy were intermediate between those observed in control animals and in those treated with cisplatin alone and were statistically different from neither. In line with these findings, mice treated with cisplatin alone showed a marked loss of body weight over the course of the study, compared with untreated BBN‐exposed animals (Fig. 5G). Importantly, this pro‐cachectic effect was significantly reduced in mice treated with the combination therapy. No differences were observed concerning food and water consumption between groups (Fig. S2). To determine if the combination therapy could preserve skeletal muscle mass, we compared the gastrocnemius muscle weights between groups, as a percentage of body weight (Fig. 5H). No differences were observed, suggesting that weight loss affected various types of tissues rather than selectively targeting skeletal muscle. We also employed a grip strength test to study the impact of cisplatin therapy on muscle function, and whether this could be rescued by DMAPT. There were no significant differences concerning maximum grip strength between groups, after normalizing by bodyweight (Fig. 5I). However, BBN exposure induced a moderate increase in muscle fatigue, which reached marginal statistical significance (*P* = 0.046, Student's *t* test) in cisplatin‐exposed animals and was again reduced by the combination therapy (Fig. 5J).

## 4 Discussion

The clinical use of agents with NF‐κB activity is currently restricted to hematological malignancies such as multiple myeloma where proteasome inhibitors such as bortezomib and related drugs block NF‐κB signaling among other targets [31]. However, these drugs show significant toxicities, pleiotropic effects, and little efficacy against solid tumors, and the wider application of NF‐κB inhibitors in the broader field of oncology remains an unmet need. One way to achieve greater specificity is to employ drugs from the sesquiterpene lactone family, of which parthenolide is possibly the best‐known example. This class of compounds selectively targets cysteine 38 on the p65 NF‐κB subunit while also inhibiting the upstream IκB kinase [32, 33]. A semi‐synthetic modification of parthenolide yielded DMAPT, which shows good oral bioavailability and single‐agent activity against leukemia cells [34]. DMAPT also sensitizes tumor cells to chemotherapeutic agents such as gemcitabine, and to radiotherapy [35, 36]. In the present study, we report three findings that support approaches to inhibit NF‐κB signaling in epithelial malignancies such as urothelial carcinoma. First, NF‐κB signaling is increased following the exposure of MIBC to platinum chemotherapy; second, the NF‐κB‐inhibitor DMAPT chemosensitizes urothelial cancer to cisplatin therapy; and third, DMAPT treatment reduces cisplatin‐induced nephrotoxicity and cachexia.

The claudin‐low MIBC subtype is characterized by constitutively high NF‐κB signaling and a particularly poor prognosis with lower overall survival when compared with other subtypes, regardless of their treatment regimen [7, 8]. GSEA analysis confirmed that platinum‐naive, claudin‐low MIBCs showed NF‐κB up‐regulation compared with other subtypes. We confirmed that an NF‐κB transcriptional signature was associated with reduced survival, providing a rationale for testing the hypothesis that pharmacological NF‐κB inhibition will improve outcomes. We have previously shown that DMAPT has modest single‐agent activity against bladder cancer cell lines [10] and now demonstrate that DMAPT enhances cisplatin effects *in vitro* and *in vivo* including marked inhibition of carcinogen‐induced bladder cancer. This was associated with significantly reduced expression of downstream secreted NF‐κB markers IL‐6 and WNT16 but only a non‐significant increase in 53BP1 foci. In another study, DMAPT was shown to inhibit DNA repair via non‐homologous end joining in non‐small‐cell lung cancer, leading to robust accumulation of 53BP1 foci [36]. The present data may suggest that, in addition to inhibition of NF‐κB signaling, inhibition of DNA repair via non‐homologous end joining could play a role in sensitizing MIBC to cisplatin but are insufficient to draw conclusions regarding this point.

We next evaluated toxicity in the BBN mouse bladder cancer model. Nephrotoxicity is a frequent and dose‐limiting side‐effect of cisplatin therapy in cancer patients, and we hypothesized that it could be reduced by DMAPT. NF‐κB inhibitors and parthenolide in particular have been shown to protect against cisplatin‐induced nephrotoxicity [29, 37]. We determined that DMAPT prevented the renal histological damage elicited by cisplatin alone; glomerular damage and leukocytic infiltration were greatly reduced although tubular damage remained. The preservation of renal morphology also had functional consequences, as the combination therapy reduced blood urea and creatinine levels. It is plausible that tubular damage is due to the direct effects of cisplatin resorption over tubular cells, while interstitial nephritis is driven by soluble factors secreted in response to initial cisplatin damage [38]. In a mouse model of acute cisplatin toxicity, we observed a limited response of NF‐κB‐regulated genes, where only *Cxcl1* was up‐regulated by cisplatin but not the DMAPT plus cisplatin combination. This could reflect the cisplatin dosing scheme and sample collection time but may also suggest that DMAPT recruits additional nephroprotective mechanisms. In fact, recent data show that parthenolide has anti‐apoptotic effects over podocytes from renal glomeruli [39], potentially explaining the reduced glomerular damage we observed. These promising results warrant a more detailed study of the mechanisms underlying DMAPT's protective activity. Our observations agree with a previous report concerning parthenolide, DMAPT's parent compound [29]. The improved pharmacokinetic properties of DMAPT over parthenolide make it possible to consider it as a realistic option to minimize cisplatin‐induced nephrotoxicity.

Combining cisplatin with an inhibitor of NF‐κB has the potential for immunological and hematological toxicity because of the essential role played by NF‐κB in the maturation and function of many cell types, particularly lymphocytes [40, 41]. We did observe near‐significant lymphopenia in animals treated with DMAPT alone, but there were no differences in circulating leukocyte counts between mice treated with cisplatin alone and those treated with combination therapy. When analyzing the maturation of lymphoid populations in the bone marrow, spleen, and thymus, we did not observe differences between mice treated with cisplatin alone or the combination therapy. These results are in line with previous reports showing that DMAPT selectively targets neoplastic cells while sparing normal lymphoid cells [34]. The mechanisms underlying this selectivity deserve additional in‐depth studies, but one likely explanation is that DMAPT preferentially targets the NF‐κB p65 subunit, while allowing other branches of this pathway to function and avoid the pleiotropic effects associated with less specific NF‐κB inhibitors. Although the canonical NF‐κB pathway is essential for lymphocyte biology, the c‐REL subunit seems to be more critically involved than p65 in B cell proliferation and maturation [42]. In leukemia patients, DMAPT showed no toxicities in doses that reduced NF‐κB in circulating blastic cells.

Dimethylaminoparthenolide has also been suggested to show protective effects against cancer cachexia, by inhibiting NF‐κB signaling. The involuntary weight loss associated with cancer, involving the loss of skeletal muscle and adipose tissues and the impairment of muscle function, is aggravated by platinum therapy [43]. The NF‐κB signaling pathway contributes to these pathologies and seems to be mediated by elevated IL‐6 serum levels [41, 44]. In our experiments, cisplatin did cause substantial weight loss and increased muscle fatigue in association with elevated IL‐6 serum levels, which agrees with the known connection between NF‐κB signaling and cachexia. Interestingly, other markers like ghrelin and myostatin remained stable, further implicating IL‐6 and NF‐κB signaling as key mediators of cancer cachexia. Our cytokine panel was not comprehensive, and other NF‐κB and non‐NF‐κB markers might help elucidate the roles of cisplatin and DMAPT in cachexia in the BBN mouse model. In this model, cisplatin‐induced cachexia seems to be characterized by weight loss affecting multiple types of tissues without necessarily inducing sarcopenia. Additional studies should focus on multiple muscles to clarify the changes occurring in muscle tissue in this model. The addition of DMAPT reduced circulating IL‐6 and prevented weight loss, suggesting it may be beneficial for cancer patients undergoing platinum therapy.

The main limitations of this study are related to the BBN mouse model, which recapitulates the basal MIBC subtype [27, 45]. Models that recapitulate the luminal subtype may complement the present results [46]. Our experiments followed a chemopreventive rather than chemotherapeutic strategy because this approach was anticipated to cause excessive weight loss and mortality. Also, the experiments did not include the possibility of studying tumor recurrence following the withdrawal of chemotherapy. A wider cytokine panel would be useful to further elucidate the mechanisms underpinning cisplatin and DMAPT roles in cachexia in the BBN mouse model.

## 5 Conclusions

Overall, this study determined that DMAPT effectively enhances platinum efficacy in a mouse model of MIBC by targeting the NF‐κB pathway, which is associated with chemoresistance in urothelial cancer. The combination partly prevented the nephrotoxic and pro‐cachectic effects of cisplatin and did not worsen immuno‐hematological toxicity (Fig. 5K). In conclusion, our work further implicates the importance of NF‐κB in urothelial cancer biology and chemoresistance. The findings that DMAPT, an agent that blocks NF‐κB, augments cisplatin activity and limits cisplatin toxicity support combining DMAPT with cisplatin in clinical trials.

## Conflict of interest

Christopher Sweeney holds a patent for parthenolide use in cancer and stock in Leuchemix which is developing DMAPT for clinical use.

## Author contributions

Conception and design: RMGC, CJS, and PSN. Development of methodology: PAO, RF, MV, JM, PC, CL, NPC, AM‐P, and RMGC. Acquisition of data (performed experiments, acquired and managed patients, provided animals and facilities, etc.): MMSMB, PAO, CL, HC, TN, RF, MV, CJS, PF, PC, AF‐R, JLW, PCB, CJS, and PSN. Analysis and interpretation of data: IMC, BC, JBC, DB‐F, H‐ML, and RMGC. Writing, review, and/or revision of the manuscript: RMGC, MMSMB, PAO, CL, HC, RF, MV, JM, PC, CL, NPC, AM‐P, CJS, PF, AF‐R, IC, BC, JBC, DB‐F, H‐ML, JLW, PCB, CJS, and PSN.

### Peer review

The peer review history for this article is available at https://www.webofscience.com/api/gateway/wos/peer‐review/10.1002/1878‐0261.13504.

## Supporting information


**Fig. S1.** DMAPT does not influence the numbers of lymphoid precursors in the bone marrow, thymus, and spleen.

**Fig. S2.** Weekly evolution of food and water consumption and body weights during the BBN experimental protocol.

**Table S1.** Primer sequences used for qRT‐PCR.

**Table S2.** Combination index values for each drug combinations using metabolic activity and viability data from UMUC‐3 and HTB9 cells.

**Table S3.** Expression of NF‐κB‐regulated genes in renal tissues of mice treated with cisplatin, DMAPT, and their combination.

## Data Availability

The transcriptome data that support the findings of this study are openly available in the Gene Expression Omnibus repository (GEO) at https://www.ncbi.nlm.nih.gov/geo/, reference number GSE87304. The survival data that support the findings of this study are available in the cBioPortal for Cancer Genomics at https://www.cbioportal.org/, reference Bladder Cancer (TCGA, Cell 2017).
