# Supplementary figures and images for: Pharmacological NF‐κB inhibition decreases cisplatin chemoresistance in muscle‐invasive bladder cancer and reduces cisplatin‐induced toxicities
Source: Mol Oncol. 2023 Sep 20;17(12):2709–27. doi: 10.1002/1878-0261.13504 (PMC10701775; doi:10.1002/1878-0261.13504)

# Supplementary figure 1

## Bone marrow

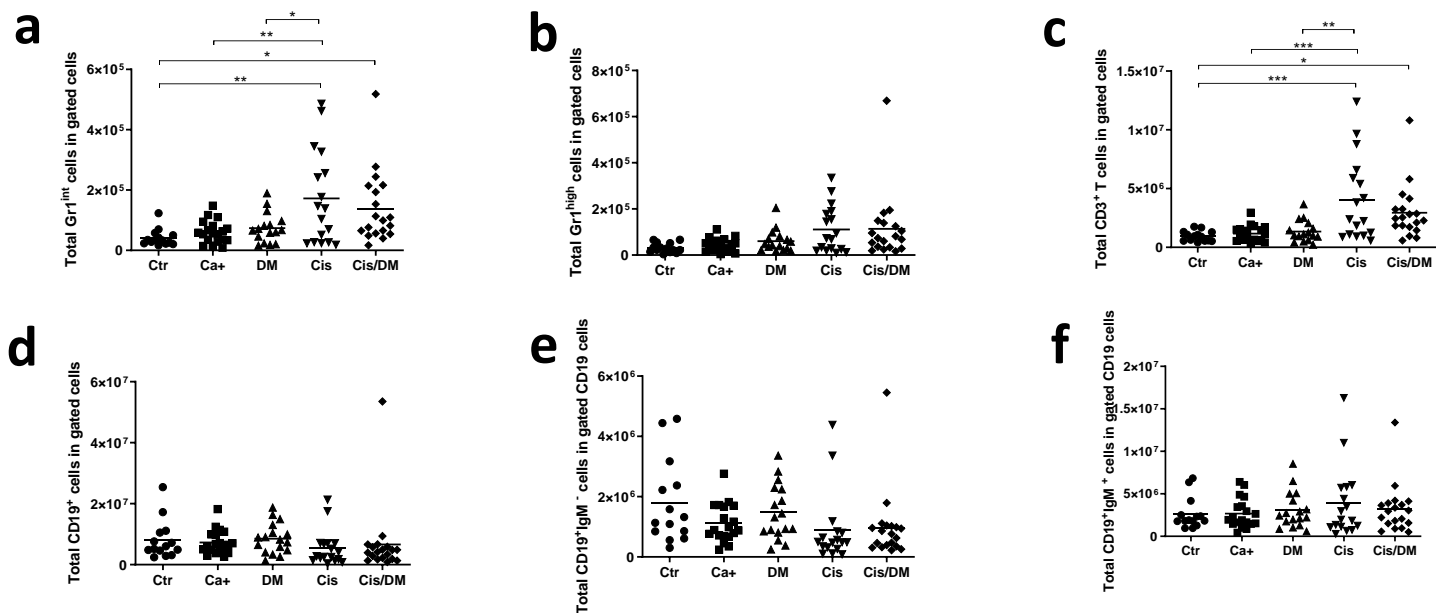

## Thymus

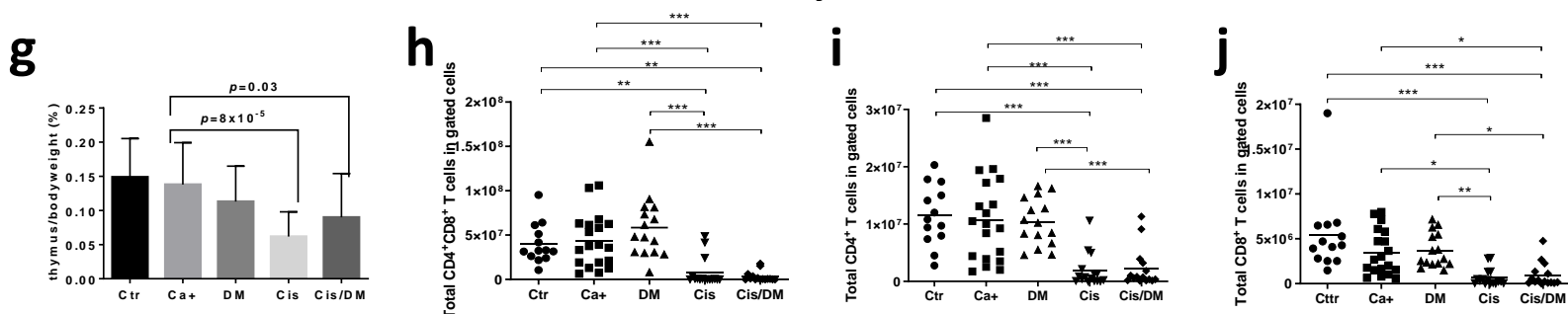

## Spleen

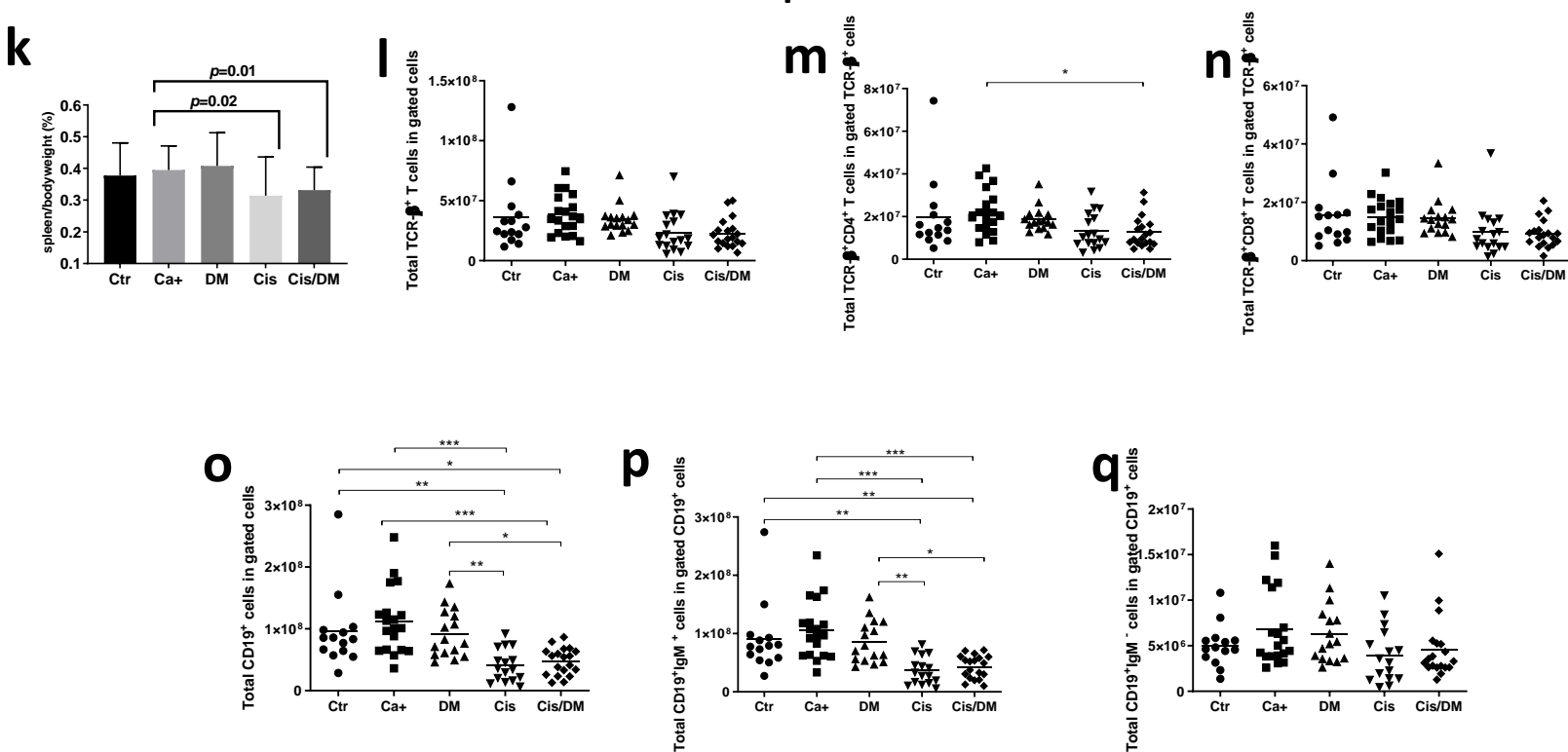

Supplement: Supplementary file 1 — Fig. S1. DMAPT does not influence the numbers of lymphoid precursors in the bone marrow, thymus, and spleen. [file MOL2-17-2709-s004.pdf]

Supplementary figure 2

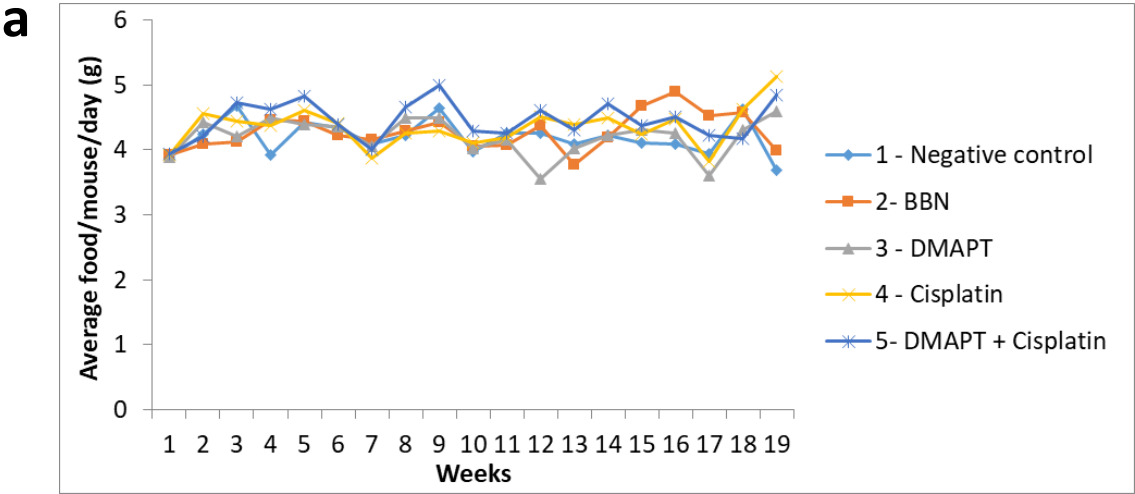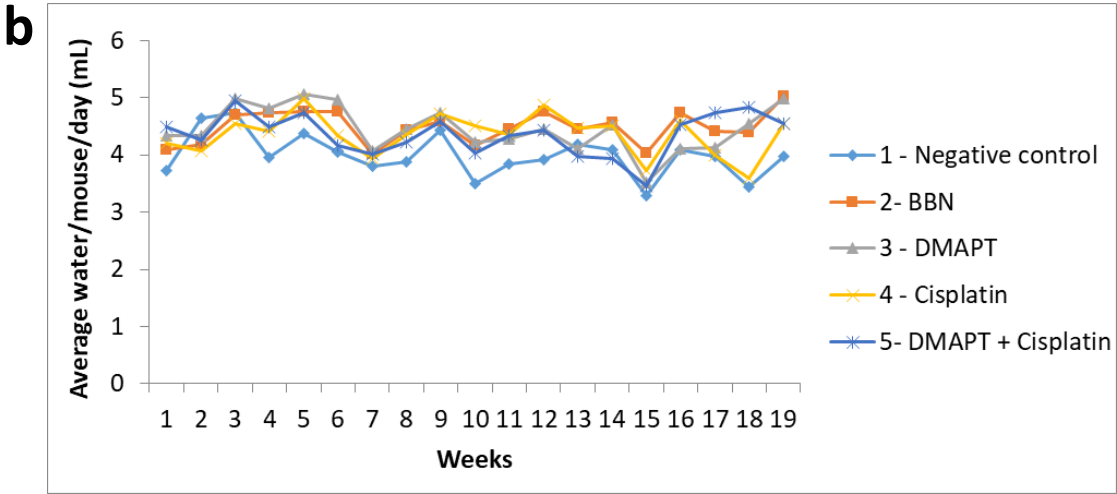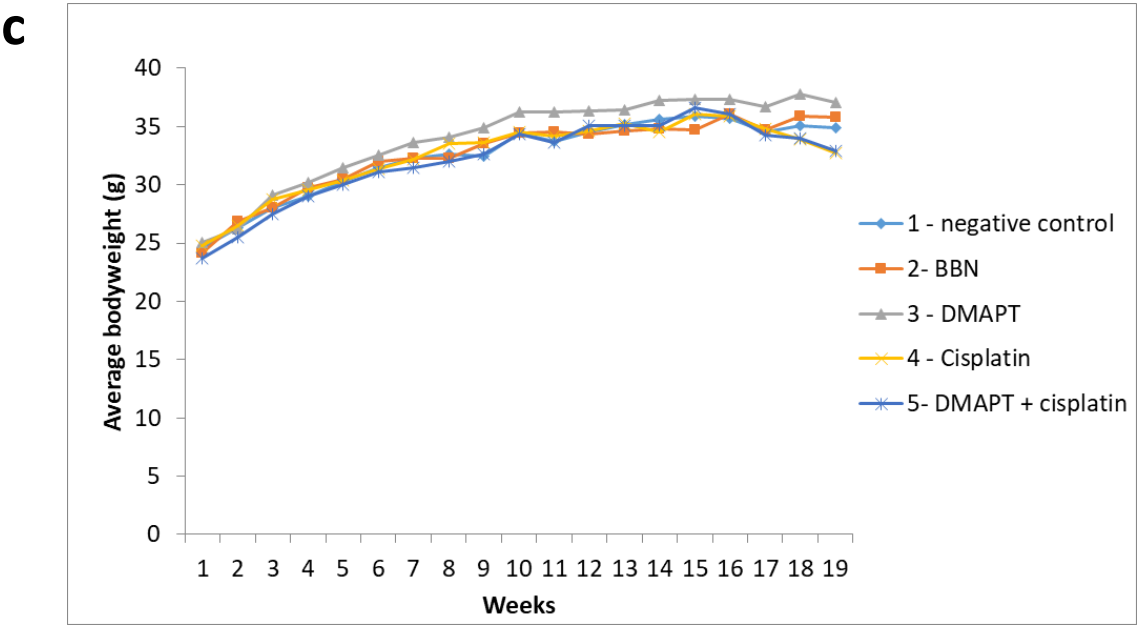

Supplement: Supplementary file 2 — Fig. S2. Weekly evolution of food and water consumption and body weights during the BBN experimental protocol. [file MOL2-17-2709-s001.pdf]
