# Supplementary material for: Pharmacological NF‐κB inhibition decreases cisplatin chemoresistance in muscle‐invasive bladder cancer and reduces cisplatin‐induced toxicities
Source: Mol Oncol. 2023 Sep 20;17(12):2709–27. doi: 10.1002/1878-0261.13504 (PMC10701775; doi:10.1002/1878-0261.13504)
Supplement: Supplementary file 3 — Table S1. Primer sequences used for qRT‐PCR. [file MOL2-17-2709-s005.docx]

Supplementary table 1 – Primer sequences used for qRT-PCR

| **Primer name** | **Sequence** |
| --- | --- |
| Cxcl1-F | GTG CCA TCA GAG CAG TCT GT |
| Cxcl1-R | GCA CCC AAA CCG AAG TCA TA |
| Cxcl5-F | GCA TTC CGC TTA GCT TTC TT |
| Cxcl5-R | GCC CTA CGG TGG AAG TCA TA |
| Il1-a-F | GGGAAGATTCTGAAGAAGAG |
| Il1-a-R | GAGTAACAGGATATTTAGAGTCG |
| Il1-b-F | TTG TTG ATG TGC TGC TGT GA |
| Il1-b-R | TGT GAA ATG CCA CCT TTT GA |
| Il6-F | CTT CAC AAG TCG GAG GCT TAA |
| Il6-R | GCA AGT GCA TCA TCG TTG TTC |
| Wnt16-F | TTT TCC AGC AGG TTT TCA CA |
| Wnt16-R | AGC AGA GTC CTG CTA GCC AT |
| Il10_F2 | GGCCCTTTGCTATGGTGTCC |
| Il10_R2 | TGGTTTCTCTTCCCAAGACCC |
| IFNg-F | TTCTTCAGCAACAGCAAGGC |
| IFNg-R | TCAGCAGCGACTCCTTTTCC |
| Tnf_F1 | GCACCACCATCAAGGACTCA |
| Tnf_R1 | GAGGCAACCTGACCACTCTC |
| Mmp2_F1 | AGGTGTGCCAAGGTGGAAAT |
| Mmp2_R1 | ACGAGCGAAGGGCATACAAA |
| Mmp7_F1 | CACTGGGTCCTCCATTGCAT |
| Mmp7_R1 | CCCATCCACAGCACAAGGAA |
| Mmp9_F2 | TTCACCGGCTAAACCACCTC |
| MMp9_R2 | GCCCGACACACAGTAAGCAT |
| Mmp20_F2 | TACCCAACCGGATGTTGACTG |
| Mmp20_R2 | CAACAGGGCACCTTGAGCTT |
| Ccl3_F2 | TCCATGGGTCCCGTGTAGAG |
| Ccl3_R2 | GTGGCTACTTGGCAGCAAAC |
| Ccl5_F2 | CTTCCCTGTCATTGCTTGCTC |
| Ccl5_R2 | CCGAGTGGGAGTAGGGGATT |
| Ptgs2_F2 | TCCAATATTGACTGACCCAAGCA |
| Ptgs2_R2 | AAACACTTACAGGGCCTTCAAAA |
