# Supplementary material for: Pharmacological NF‐κB inhibition decreases cisplatin chemoresistance in muscle‐invasive bladder cancer and reduces cisplatin‐induced toxicities
Source: Mol Oncol. 2023 Sep 20;17(12):2709–27. doi: 10.1002/1878-0261.13504 (PMC10701775; doi:10.1002/1878-0261.13504)
Supplement: Supplementary file 4 — Table S2. Combination index values for each drug combinations using metabolic activity and viability data from UMUC‐3 and HTB9 cells. [file MOL2-17-2709-s003.docx]

Supplementary table 2 – Combination index values for each drug combinations using metabolic activity and viability data from UMUC-3 and HTB9 cells.

Synergism: CI<1, additive CI=1, antagonistic CI>1.

| **Cell lines** | **Variable** | **Combination index (CI)** | | |
| --- | --- | --- | --- | --- |
|  |  | **1:1µM** | **2.5:2.5 µM** | **5:5 µM** |
| UMUC-3 | Metabolic activity | 0.28795 | 0.56511 | 0.84751 |
| HTB9 | Metabolic activity | 0.14458 | 0,23377 | 0,34036 |
| UMUC-3 | Viability | 0,39181 | 0,83168 | 1,18526 |
| HTB9 | Viability | 0,60336 | 1,08404 | 1,17286 |
