# Supplementary material for: Pharmacological NF‐κB inhibition decreases cisplatin chemoresistance in muscle‐invasive bladder cancer and reduces cisplatin‐induced toxicities
Source: Mol Oncol. 2023 Sep 20;17(12):2709–27. doi: 10.1002/1878-0261.13504 (PMC10701775; doi:10.1002/1878-0261.13504)
Supplement: Supplementary file 5 — Table S3. Expression of NF‐κB‐regulated genes in renal tissues of mice treated with cisplatin, DMAPT, and their combination. [file MOL2-17-2709-s002.docx]

Supplementary table 3 – Expression of NF-κB-regulated genes in renal tissues of mice treated with cisplatin, DMAPT and their combination.

|  | **Average ΔCT (S16) ± STD** | | | | |
| --- | --- | --- | --- | --- | --- |
| **gene** | **Ctr** | **Cis4** | **Cis4DM** | **Cis10** | **Cis10DM** |
| **24 hours** | | | | | |
| ***Cxcl1*** | 6.059 ± 0.658 | 6.894 ± 0.810 | 6.863 ± 0.287 | 4.199 ± 0.837 | 6.156 ± 0.370 |
| ***Cxcl5*** | 11.874 ± 1.480 | 13.348 ± 2.857 | 12.069 ± 1.025 | 11.276 ± 1.559 | 12.870 ± 0.355 |
| ***Il1a*** | 17.954 ± 0.904 | 18.613 ±0.820 | 18.368 ± 1.013 | 19.097 ± 1.984 | 19.653 ± 0.647 |
| ***Il1b*** | 7.386 ± 0.824 | 7.345 ± 0.421 | 8.025 ± 0.644 | 7.180 ± 0.734 | 8.467 ± 0.216 |
| ***Il6*** | 12.114 ± 1.468 | 12.344 ± 0.780 | 12.404 ± 0.804 | 12.786 ± 1.125 | 12.947 ± 0.198 |
| ***Il10*** | 10.242 ± 1.118 | 10.175 ± 0.963 | 9.982 ± 1.243 | 10.058 ± 0.791 | 10.550 ± 0.522 |
| ***Tnf*** | 8.896 ± 0.998 | 8.939 ± 0.365 | 9.013 ± 0.590 | 8.651 ± 0.554 | 9.360 ± 0.724 |
| ***Ifng*** | 10.867 ± 0.953 | 10.672 ± 0.961 | 11.070 ± 1.101 | 11.135 ± 0.481 | 11.794 ± 0.390 |
| ***Wnt16*** | 12.833 ± 0.392 | 11.342 ± 0.657 | 12.241 ± 0.549 | 11.685 ± 0.866 | 12.443 ± 0.296 |
| ***Ccl3*** | 9.108 ± 1.149 | 9.097 ± 0.387 | 8.722 ± 0.433 | 9.336 ± 0.403 | 9.082 ± 1.247 |
| ***Ccl5*** | 5.661 ± 1.261 | 5.882 ± 0.481 | 5.533 ± 0.813 | 5.055 ± 0.435 | 6.438 ± 0.285 |
| ***Mmp2*** | 6.329 ± 0.484 | 6.357 ± 0.261 | 6.449 ± 0.291 | 6.722 ± 0.591 | 6.386 ± 0.630 |
| ***Mmp7*** | 7.009 ± 1.329 | 6.922 ± 2.004 | 7.172 ± 1.073 | 6.696 ± 1.384 | 6.398 ± 0.281 |
| ***Mmp9*** | 9.267 ± 0.422 | 9.481 ± 0.429 | 9.205 ± 0.296 | 9.431 ± 0.990 | 9.278 ± 2.129 |
| ***Mmp20*** | 12.544 ± 1.122 | 12.586 ± 0.531 | 12.172 ± 1.863 | 11.689 ± 0.472 | 11.483 ± 0.916 |
| ***Ptgs2*** | 7.336 ± 2.311 | 7.728 ± 2.181 | 8.443 ± 1.381 | 6.804 ± 2.899 | 7.042 ± 0.160 |
| **2 weeks** | | | | | |
| ***Cxcl1*** | 5.919 ± 0.857 | 6.751 ± 0.593 | 6.408 ± 0.737 | 6.384 ± 0.619 | 5.221 ± 0.611 |
| ***Cxcl5*** | 9.317 ± 1.757 | 10.707 ± 1.160 | 11.275 ± 1.012 | 9.818 ± 2.731 | 12.870 ± 1.315 |
| ***Il1a*** | 17.670 ± 2.547 | 15.441 ± 3.667 | 18.510 ± 1.295 | 17.457 ± 1.313 | 19.653 ± 1.320 |
| ***Il1b*** | 6. 164 ± 0.592 | 6.648 ± 0.696 | 7.439 ± 0.644 | 6.661 ± 1.078 | 7.352 ± 0.216 |
| ***Il6*** | 11.022 ± 1.290 | 10.797 ± 0.902 | 10.949 ± 1.264 | 10.720 ± 1.371 | 10.028 ± 1.074 |
| ***Il10*** | 9.881 ± | 9.639 ± 1.138 | 9.609 ± 1.086 | 10.388 ± 0.709 | 9.097 ± 0.888 |
| ***Tnf*** | 8.056 ± | 8.260 ± 0.701 | 8.657 ± 0.601 | 8.744 ± 0.574 | 7.703 ± 0.642 |
| ***Ifng*** | 10.427 ± 0.774 | 10.338 ± 0.972 | 10.447 ± 1.198 | 11.124 ± 0.552 | 10.547 ± 1.347 |
| ***Wnt16*** | 12.010 ± 0.492 | 11.949 ±0.624 | 12.262 ±0.309 | 11.987 ± 0.256 | 12.316 ± 0.294 |
| ***Ccl3*** | 8.244 ± | 8.389 ± 0.869 | 8.702 ± 0.750 | 8.879 ± 1.174 | 7.759 ± 0.691 |
| ***Ccl5*** | 5.069 ± | 4.743 ± 0.972 | 4.970 ± 0.988 | 5.493 ± 0.651 | 3.396 ± 1.151 |
| ***Mmp2*** | 5.459 ± | 6.125 ± 0.105 | 5.600 ± 0.168 | 5.456 ± 0.472 | 4.490 ± 0.235 |
| ***Mmp7*** | 7.327 ± | 6.963 ± 1.022 | 7.950 ± 1.062 | 8.740 ± 2.268 | 5.985 ± 1.216 |
| ***Mmp9*** | 8.235 ± | 8.302 ± 0.269 | 8.415 ± 0.497 | 8.516 ± 1.003 | 7.126 ± 0.483 |
| ***Mmp20*** | 11.976 ± | 12.186 ± 1.588 | 11.788 ± 0.685 | 11.735 ± 0.751 | 11.234 ± 0.181 |
| ***Ptgs2*** | 6.764 ± | 8.035 ± 1.190 | 7.544 ± 1.385 | 7.104 ± 1.877 | 6.217 ± 1.824 |

(Ctr - negative control, Cis4 - 4 mg/kg cisplatin, Cis4DM - 4 mg/kg cisplatin and 100 mg/kg DMAPT, Cis10 - 10 mg/kg cisplatin, Cis10DM - 10 mg/kg cisplatin and 100 mg/kg DMAPT).
